# Serum Biomarkers of Liver Fibrosis Staging in the Era of the Concept “Compensated Advanced Chronic Liver Disease”

**DOI:** 10.3390/jcm10153340

**Published:** 2021-07-28

**Authors:** Koji Fujita, Tsutomu Masaki

**Affiliations:** Department of Gastroenterology and Neurology, Faculty of Medicine, Kagawa University, 1750-1 Ikenobe, Miki, Kita District, Kagawa 761-0793, Japan; tmasaki@med.kagawa-u.ac.jp

**Keywords:** biomarkers, biopsy, needle, diagnosis, liver cirrhosis, portal hypertension

## Abstract

Non-invasive indexes of liver fibrosis based on blood examinations have been developed for decades, partially replacing liver biopsy examinations. Recently, the concept of liver cirrhosis was revised and converted to “compensated advanced chronic liver diseases” since the Baveno VI consensus statement in 2015. The term “compensated advanced chronic liver diseases” was established based on the premise that serum biomarkers were not able to differentiate cirrhosis from severe fibrosis. The difficulty to histologically distinguish cirrhosis from severe fibrosis had been pointed out in 1977, when the definition and nomenclatures of cirrhosis had been determined by the World Health Organization. That was decades before serum biomarkers available at present were investigated. Though we are accustomed to differentiating the fibrosis stage as stage 1, 2, 3 (severe fibrosis), and 4 (cirrhosis), differentiation of cirrhosis from severe fibrosis is difficult even by histopathological examination. The current review will provide readers a framework to revise how to apply serum biomarkers on liver fibrosis staging in an era of the concept of “compensated advanced chronic liver disease”.

## 1. Introduction

Recently, a consensus statement concerning portal hypertension and esophageal varices confirmed that distinguishing cirrhosis from noncirrhotic status was often not possible on clinical grounds [1]. The statement is called the Baveno VI consensus, which focused on the detection of esophageal varices and the prevention of its rupture. The consensus emphasizes the clinical significance to detect portal hypertension in its early phase through transient elastography, instead of the traditional method, to determine the liver fibrosis stage. In addition, the study group proposes the concept of compensated advanced chronic liver diseases (cACLDs) by removing the borderline between the cirrhosis and noncirrhotic status.

Non-invasive indexes of liver fibrosis based on blood examinations have been developed for decades, partially replacing liver biopsy examinations [2]. Since the prevalence of hepatocellular carcinoma (HCC) and esophageal varices correlates to the progression of the fibrosis stage [3,4], the indexes provide important suggestions for physicians and liver specialists as alternatives that are easy to handle compared to pathological diagnosis [5,6]. The strengths of serum biomarkers include high reproducibility, easy availability since they are not protected by patents, outpatient diagnosis, and adequate validation by accumulated evidence [7].

Serum biomarkers were developed based on histological scoring systems including METAVIR scoring systems [8]. Scoring systems were designed to differentiate cirrhosis from noncirrhotic status. However, the difficulty to classify advanced liver fibrosis clearly into severe fibrosis (stage 3) and cirrhosis (stage 4) had been pointed out since 1977 [9,10]. Although the classification of liver fibrosis is familiar to clinicians, differentiating cirrhosis from severe fibrosis has been difficult even by histopathological examination. Pathological staging is an imperfect gold standard for liver fibrosis, so even a perfect surrogate may not be sufficient to achieve perfect diagnostic accuracy [11].

In the current review, the significance and limitation of non-invasive indexes of liver fibrosis based on blood examinations were reviewed, concerning the revision of the classical idea “liver cirrhosis”.

## 2. Liver Biopsy Examination

Liver biopsy has been the gold standard in the evaluation of the liver fibrosis stage [12] because the ideas of fibrosis and cirrhosis originated from pathology [13,14]. Liver biopsy examinations detect the fibrotic change in biopsy specimens, usually that of the right hepatic lobe [15].

A tissue obtained by a needle biopsy might be 1/50,000 or less in its volume compared to a whole liver [15]. Fibrosis progression is not homogeneous in the liver. By percutaneous liver biopsy, the difficulty to differentiate cirrhosis from chronic active hepatitis was addressed when the terminology of cirrhosis was determined in 1977 [9,10]. Another report pointed out the discrepancy of liver fibrosis stages between the right and left lobes [16]. The sample size should be adequate for pathological evaluation. If a sample is too small, the pathological decision might be more difficult and inaccurate [17,18]. While pathological findings should be interpreted and categorized to classify the fibrosis stage according to the conventional definition, the interpretation is subject to intraobserver and interobserver variations [16,19,20]. Overall, considering sampling error and intra- and interobserver variability, the discrepancy rate in fibrosis staging can be as high as 25%, even for a 25-mm-long specimen [18]. Furthermore, serious complications including bleeding are also recognized [21]. Noninvasive biomarkers based on blood examinations have been, thus, investigated to evade the problems mentioned above.

**Figure 1:**
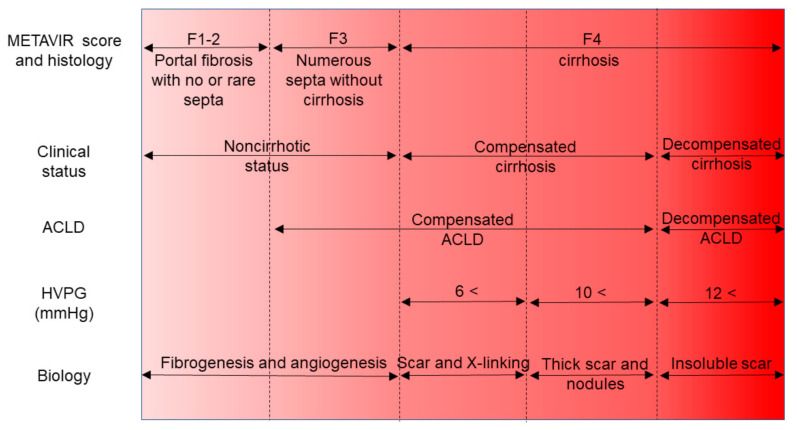
Contrast of the fibrosis stage, clinical definition, and ACLD.

As a representative classification of the fibrosis stage, the histology of the METAVIR scoring system is summarized in Figure 1. The definition of each stage is as follows: F0, no fibrosis; F1, portal fibrosis without septa; F2, portal fibrosis with rare septa; F3, numerous septa without cirrhosis; F4, cirrhosis [8,22]. Severe fibrosis means F3. Another classification proposed by Desmet et al. defined F2 as porto-portal septa and F3 as portocentral septa [23].

Differentiation of chronic liver disease is based on the METAVIR scoring system, clinical classification, portal hypertension, and representative characteristics in histology. The non-cirrhotic stage (METAVIR F1-F3) is defined as a series of statuses with no clinical evidence of cirrhosis. The cirrhosis (METAVIR F4) ranged from compensated to decompensated stages with life-threatening complications including hepatocellular carcinoma, liver failure, and esophageal varices rupture. The concept of ACLD covers F3 and F4 based on the premise that clinical differentiation of cirrhosis from severe fibrosis is often difficult. Instead of histological classification, clinical manifestation divides ACLD into compensated and decompensated types. In the context of HVPG measurements, the threshold pressure >10 mmHg predicts the development of varices. An HVPG of >10 mmHg correlates with a thick septa and small nodules. In the decompensated stage, the septa resist degradation. The figure is modified from Friedman [24] and Garcia-Tsao [25]. The abbreviation definitions are: ACLD; advanced chronic liver diseases; HVPG, hepatic venous pressure gradient

## 3. Serum Biomarkers for Liver Fibrosis

Fibrosis indexes based on blood examination have been classified as direct and indirect ones according to their origin. The former consists of byproducts or cytokines produced and released in the dynamics of extracellular matrix synthesis versus degradation predominantly performed by activated hepatic stellate cells [26]. The latter reflects the consequence of fibrosis progression including injury of hepatocyte and hypersplenism. The elevation of alanine transaminase (ALT) and decrease of platelet count represent preferable parameters of indirect indexes [27,28].

Notably, it does not naturally mean that direct indexes are superior to indirect ones in the diagnosis of the fibrosis stage [29]. Both categories of fibrosis indexes seem to need prior cut-off values specific to etiology. For major biomarkers, their diagnostic abilities to diagnose advanced fibrosis and cirrhosis are presented as the area under the receiver operating characteristic (ROC) curve (95% confidence interval) and case number of the cohort that each study was based on (Table 1).

Diagnostic accuracy of serum biomarkers is presented by the area under the ROC curve with the 95% confidence interval and case number. The abbreviation definitions are: FIB-4, fibrosis-4 index; APRI, AST to platelet ratio index; WFA^+^-M2BP, Wisteria floribunda agglutinin-positive Mac-2 binding protein; ELF score, enhanced liver fibrosis score.

### 3.1. The Mechanisms of Liver Fibrosis with a Focus on Fibrosis Biomarkers

**Figure 2:**
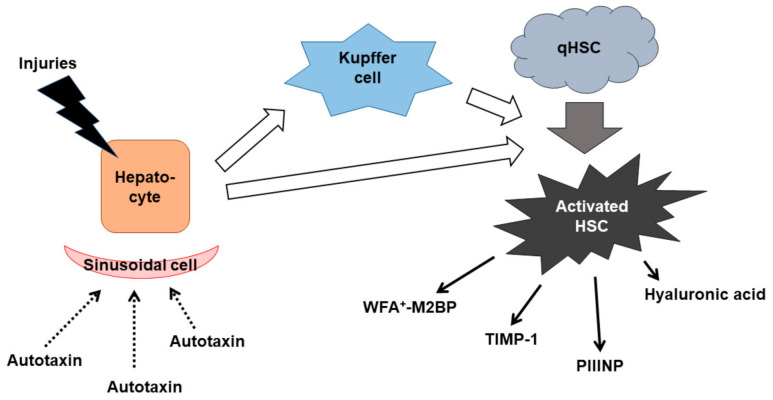
Mechanisms of liver fibrosis with a focus on fibrosis biomarkers.

A direct type of fibrosis indicator relates to the mechanism of liver fibrogenesis. Liver fibrosis consists of the production of the extracellular matrix by activated hepatic stellate cells (Figure 2) [44]. Different types of injuries, such as infections, drugs, metabolic disorders, and immune attacks, cause inflammation in the liver tissue. Inflammation activates Kupffer cells, which are liver-specific macrophages. Cytokines secreted by hepatocytes, and Kupffer cells activate hepatic stellate cells. Activated hepatic stellate cells produce extracellular matrix molecules and cytokines such as hyaluronic acid, type III collagen, tissue inhibitor of metalloproteinases-1 (TIMP-1), and the Wisteria floribunda agglutinin-positive Mac-2 binding protein (WFA^+^-M2BP). Aminoterminal propeptide of type III procollagen (PIIINP) is one of the metabolites derived from type III procollagen.

When stimulated by a variety of causes, including infection, toxins, metabolic abnormalities, and altered immune responses, hepatocytes secrete cytokines that cause tissue inflammation. Kupffer cells are also activated and secrete proinflammatory cytokines. As a result, quiescent hepatic stellate cells are activated and produce extracellular matrix molecules and proinflammatory cytokines. Liver inflammation and fibrosis lead to dysfunction of hepatic sinusoidal epithelial cells. The catabolism of autotaxin by hepatic sinusoidal epithelial cells is inhibited by the progression of hepatic fibrosis.

The abbreviation definitions are: qHSC, quiescent hepatic stellate cell; WFA^+^-M2BP, Wisteria floribunda agglutinin-positive Mac-2 binding protein; TIMP-1, tissue inhibitor of metalloproteinases-1; PIIINP, aminoterminal propeptide of type III procollagen

### 3.2. Fibrosis-4 Index

The fibrosis-4 index is one of the most widely applied indirect biomarkers of liver fibrosis [45]. The index is constructed by age, aspartate aminotransferase (AST), ALT, and platelet count: fibrosis-4 index = age × AST (U/l)/(Plt (10^9^/l) × √ALT (U/l)) [46]. Cut-off values and their diagnostic abilities for liver fibrosis are described for each etiology of background liver diseases including hepatitis C virus (HCV) [47], hepatitis B virus (HBV) [48], and non-alcoholic fatty liver disease [49].

Since age is incorporated in the index, the fibrosis-4 index might overestimate the fibrosis stage in senior patients [50]. Recently, regression of fibrosis severity following antiviral therapy was highlighted [51], as HCV infection is being cured by direct antiviral agents [52], and activity of HBV infection is under control by nucleos(t)ide analogs [53]. The application of the fibrosis-4 index on senior patients after antiviral therapy might be assessed further.

### 3.3. AST to Platelet Ratio Index

The AST to platelet ratio index (APRI) is also one of the major indirect indexes composed of AST and platelet count: APRI = 100 × AST (U/l)/upper limit of normal AST values (U/l)/Plt (10^9^/l) [45]. The clinical significance of APRI was established in a cohort with HCV infection [28]. APRI assessed the diagnostic accuracy of liver fibrosis compared to that of the fibrosis-4 index in HBV infection [54]. APRI can be applied for nonalcoholic fatty liver diseases as an alternative to the fibrosis-4 index [49].

### 3.4. FibroTest

The FibroTest was investigated to diagnose significant fibrosis (F2-4) in HCV infection [55]. The score is constructed by a panel of five items in biochemistry calculated based on the equation: f = 4.467 × log (alpha-2-macroglobulin (g/L)) − 1.357 × log (haptoglobin (g/L)) + 1.017 × log (γ-glutamyl transpeptidase (IU/L)) + 0.0281 × age + 1.737 × log (bilirubin (µmol/L)) − 1.184 × (apolipoprotein A1 (g/L)) + 0.301 × sex (female 0, male 1) − 5.540. Application of the score on other etiologies were validated including HBV [43], NASH [36], and alcoholic liver disease [56].

### 3.5. WFA^+^-M2BP

The WFA^+^-M2BP was invented as a serum glycobiomarker for the assessment of liver fibrosis in Japan [57]. M2BP, one of the multibranching and sialylated N-glycans, is modified with a fibrosis-specific sugar chain in chronic liver diseases [58]. WFA^+^-M2BP is, thus, classified as a direct biomarker (Figure 2). The serum level of M2BP is determined by WFA that recognizes the N-acetylgalactosamine residue of N-glycans and O-glycans on M2BP [59]. Serum WFA^+^-M2BP levels were originally reported not to correlate with the severity of hepatitis activity [59,60]. However, the correlation between WFA^+^-M2BP and hepatic inflammation was suggested in another study [61].

### 3.6. Enhanced Liver Fibrosis Score

Similar to WFA^+^-M2BP, the enhanced liver fibrosis (ELF) score measures components of the extracellular matrix produced by activated stellate cells in fibrotic liver in Europe [62]. The ELF score is calculated based on PIIINP, hyaluronic acid (HA) and TIMP-1, and age. PIIINP is produced from type III procollagen. PIIINP, HA, and TIMP-1 are all produced by activated hepatic stellate cells (Figure 2).

The original index was investigated in a cohort with primary biliary cholangitis [6]. The index was then applied on a pediatric cohort with nonalcoholic fatty liver disease through a revised equation omitting age from the equation: −7.412 + ln(HA) × 0.681 + ln(PIIINP) × 0.775 + ln(TIMP-1) × 0.494 + 10 [62]. The index was also adjusted to a senior cohort with HCV infection by the revised equation [30].

### 3.7. Others

In liver fibrosis, components of the extracellular matrix (ECM) are accumulated [63]. Dysregulation of ECM composition, structure, stiffness, and abundance contributes to several pathological conditions including fibrosis [64].

Type IV collagen is widely and exclusively distributed in basement membranes [65]. In the cirrhotic liver, the expression of type IV collagen increased up to 14-fold [66]. The degree of fibrosis or cell infiltration in the liver significantly correlated to the serum type IV collagen level [67].

Hyaluronic acid has been considered a biomarker for evaluating chronic liver diseases since 1985 [68]. This glycosaminoglycan with a high molecular weight, nonsulfated, linear chain is an important constituent of the extracellular matrix [69]. Hyaluronic acid is produced by hepatic stellate cells and finally degraded by sinusoidal endothelial cells in the liver [70]. Clinical application of hyaluronic acid on liver fibrosis staging is presented by a comprehensive review [71].

Autotaxin is unique for liver fibrosis as a biomarker by its physiological kinetics. Autotaxin is a secreted lysophospholipase D that catalyzes lysophosphatidylcholine to a lipid mediator, lysophosphatidic acid (LPA) [72]. LPA activates G protein-coupled receptors to evoke various cellular responses. Autotaxin is degraded by liver sinusoidal endothelial cells [73] (Figure 2). Serum autotaxin is inversely correlated to the severity of liver fibrosis [74,75]. Cut-off values of autotaxin should be greater for females than for males [76].

## 4. Revision of the Idea “Liver Cirrhosis”

Liver cirrhosis was considered important because cirrhosis was presented with life-limiting complications including HCC, liver failure, and esophageal varices rupture [77]. Cirrhosis was distinguished from other hepatic fibrosis supported by the ideas: (1) cirrhosis was in an irreversible state in most cases, (2) cirrhosis was the common status regardless of their etiologies in their end-stage, (3) cirrhosis was pathologically characterized by structurally abnormal nodules, and (4) cirrhosis meant the loss of physiological function of a liver [9,10,78].

However, therapeutic advances in the field of chronic liver diseases drastically changed the natural history of cirrhosis. The viral eradication of HCV [79] or inhibition of HBV replication [80], administration of immunosuppressive agents for an altered and excessive immune response [81], and removing offending factors including alcohol and iron [82] succeeded in the regression to liver fibrosis from cirrhotic status. Patients were able to survive with cirrhosis, even with clinically improved liver function in their time course [25]. Since then, incorporating the clinical and hemodynamic findings with histology, a pathophysiologic staging of liver fibrosis was studied [83].

### 4.1. Focus on the Hepatic Venous Pressure Gradient

In the prediction of esophageal varices development and rupture, hepatic venous pressure gradient (HVPG) was highlighted as an emerging parameter [84]. An increased HVPG level presented a good correlation with the complications of portal hypertension. Esophageal varices are complicated in patients with an HVPG of >10 mmHg [85]. When HVPG increases over 12 mmHg, varices rupture (Figure 1).

Histological features of the liver-associated with HVPG probed small parenchymal nodules and thick fibrous septa [86]. The thickness of the fibrous septa has been, thus, suggested as a tool to stage cACLD (Figure 1). Following the findings above, a validation study revealed that the degree of collagen in the space of Disse and histological grade of cirrhosis significantly correlated to high HVPG [87].

Since measurement of HVPG by the transcatheter technique was not less invasive than a liver biopsy, a non-invasive strategy to evaluate portal hypertension should replace them. Transient elastography, a non-invasive method, has been found to provide an excellent correlation with HVPG values up to a level of 10–12 mmHg in patients with chronic hepatitis C [88,89].

### 4.2. Concept of the Compensated Advanced Chronic Liver Disease

Based on accumulated evidence and several conferences on hemodynamic changes through liver fibrosis progression [90,91,92,93,94], a term, namely, cACLD was proposed in the Baveno VI consensus statement for patients at risk of developing clinically significant portal hypertension in 2015 [1]. The idea has been intended to clarify that the spectrum of severe fibrosis and cirrhosis is a continuum in asymptomatic patients and that distinguishing between the two is often not possible on clinical grounds (Figure 1).

The description in the statement confirmed the difficulty in differentiation between cirrhosis and non-cirrhotic status [1]. Cirrhosis was defined as a process in which normal liver architecture was being converted to abnormal nodules with fibrosis in the whole organ. The development of cirrhotic nodules is not simultaneously observed in any regions of the liver. Furthermore, the onset of cirrhotic change is not accurately determined. Then, cirrhosis is not clearly divided from precirrhotic status. While scoring systems of liver fibrosis including METAVIR [8] and the Ishak score [95] categorize fibrosis severity into several stages, the development of cirrhotic nodules progresses and continues gradually through the continuum of severe fibrosis and cirrhotic status.

### 4.3. Fibrosis Staging by Noninvasive Biomarkers

The differential diagnosis of F4 from F1-3 would indeed be accurate enough with the area under the ROC curve of more than 0.8 through indirect biomarkers based on blood examinations [7]. However, the dichotomized approach, the way to split a series of fibrosis progression as F1-3 vs. F4, F1-2 vs. F3-4, or F1 vs. F2-4, loses a significant amount of information [96].

Differentiation of F4 from F3 should be more difficult through a non-invasive diagnosis than a histopathological approach. In the case of transient elastography, which played definitive roles in the Baveno VI elastography criteria, the area under the ROC curve for differentiating F4 from F3 resulted in unacceptable low values [97]. The distribution of measurements of F4 also overlaps with that of F3 in direct and indirect biomarkers [28,47,57,62]. The diagnostic accuracy of non-invasive modalities concluded that they are unable to determine fibrosis progression stage by stage [12,96]. The way to apply direct and indirect biomarkers might be at the time of revision along with the establishment of the concept of “cACLD.”

A simple question for the concept “cACLD” might be raised based on ROC analysis of non-invasive diagnosis for the liver fibrosis stage. Generally, the area under the ROC in F3-4 vs. F1-2 is smaller than that of F4 vs. F1-3 by transient elastography, similar to serum biomarkers (Table 1). If the non-invasive diagnosis of F4 were to be impossible, that of F3-4 might be more difficult. It might mean that non-invasive diagnoses of cACLD should still be difficult by any modality.

Based on the Baveno VI elastography criteria, a dual liver stiffness by transient elastography threshold of <10 and >15 kPa was proposed for excluding and diagnosing cACLD in the absence of other clinical signs. For patients with liver stiffness between 10 and 15 kPa, diagnosis of cACLD might be still difficult.

The latest guideline published by EASL proposed a cut-off value of 8 kPa for liver stiffness to assist the diagnosis of advanced fibrosis [98]. However, the cut-off value premise is that the FIB-4 index is greater than 1.30. Furthermore, patients with FIB-4 >1.30 and liver stiffness > 8 kPa should be evaluated by other serum biomarkers including the ELF score and FibroTest result for validation.

### 4.4. Fibrosis Stage as a Surrogate for the Prognosis of Chronic Liver Diseases

The fibrosis stage itself should be a surrogate for clinical outcomes of chronic liver diseases [99]. While the clinical significance of surrogate markers for liver fibrosis should be determined by a clinical end-point rather than biopsy [100], the clinical significance of liver fibrosis also should be evaluated by the prognosis of patients. Patients with chronic liver diseases are complicated with HCC, end-stage liver failure, and esophageal varices rupture [101,102]. While fibrosis indexes provide the prevalence of HCC in each fibrosis stage, tumor markers are superior to fibrosis indexes in the early detection of HCC [103]. For evaluating the loss of functional hepatic reserve, alternative noninvasive indexes including the Child–Pugh classification, albumin bilirubin grade, and MELD score are available [104,105,106]. For the other major complications in cirrhosis and esophageal varices, transient elastography is emerging in the non-invasive prediction of high-risk esophageal varices [107].

## 5. Conclusions

Non-invasive indexes based on blood examinations are useful for liver fibrosis staging in clinical practice. However, the non-invasive evaluation of the fibrosis stage has limited accuracy. Following the revision of the idea of “liver cirrhosis” to “cACLD”, it might be possible to apply serum indexes at the time of revision.

## Figures and Tables

**Table 1:** Diagnostic abilities of serum biomarkers for advanced fibrosis and cirrhosis.

F3-4 vs. F1-2	HCV	HBV	NASH
FIB-4	0.87 (0.83–0.91), *n* = 340 [30]	0.91 (0.89–0.93), *n* = 699 [31]	0.80 (0.77–0.84), *n* = 8245 [32]
APRI	0.68 (-), *n*= 324 [33]	0.65 (0.56–0.73), *n* = 1521 [34]	0.75 (0.72–0.77), *n* = 6746 [32]
Fibrotest	0.73 (0.69–0.77), *n* = 504 [35]	-	0.74 (–), *n* = 452 [36]
WFA^+^-M2BP	0.78 (0.74–0.82), *n* = 1609 [37]	0.75 (0.71–0.79), *n* = 1602 [37]	0.77 (0.73–0.81), *n* = 701 [37]
ELF score	0.83 (0.79–0.87), *n* = 340 [30]	0.80 (0.73–0.87), *n* = 182 [38]	0.80 (0.80-0.80), *n* = 3173 [39]
F4 vs. F1-3			
FIB-4	0.89 (0.85-0.92), *n* = 340 [30]	0.93 (0.91–0.95), *n* = 699 [31]	0.85 (0.81–0.89), *n* = 1872 [32]
APRI	0.83 (0.78-0.88), *n*= 4266 [40]	0.75 (-), *n*= 1798 [41]	0.75 (0.70–0.80), *n* = 2196 [32]
Fibrotest	0.90 (–), *n* = 1679 [42]	0.87 (0.85–0.90), *n* = 1754 [43]	0.76 (–), *n* = 452 [36]
WFA^+^-M2BP	0.87 (0.83–0.89), *n* = 859 [37]	0.81 (0.77–0.84), *n* = 1283 [37]	0.85 (0.82–0.88) *n* = 728 [37]
ELF score	0.82 (0.78-0.87), *n* = 340 [30]	0.83 (0.76–090), *n* = 182 [38]	0.76 (0.76-0.77), *n* = 3173 [39]

## Data Availability

Not applicable.
